# Clinical outcomes of retrograde intra-arterial chemotherapy concurrent with radiotherapy for elderly oral squamous cell carcinoma patients aged over 80 years old

**DOI:** 10.1186/s13014-017-0847-3

**Published:** 2017-07-03

**Authors:** Yuichiro Hayashi, Kenji Mitsudo, Kaname Sakuma, Masaki Iida, Toshinori Iwai, Hideyuki Nakashima, Yoshiyuki Okamoto, Toshiyuki Koizumi, Senri Oguri, Makoto Hirota, Mitomu Kioi, Izumi Koike, Masaharu Hata, Iwai Tohnai

**Affiliations:** 1 Department of Oral and Maxillofacial Surgery, Yokohama City University Graduate School of Medicine, 3-9 Fukuura, Kanazawa-ku, Yokohama, Kanagawa 236-0004 Japan; 2 Department of Radiology, Yokohama City University Graduate School of Medicine, 3-9 Fukuura, Kanazawa-ku, Yokohama, Kanagawa 236-0004 Japan

**Keywords:** Intra-arterial chemotherapy, Elderly patient, Head and neck cancer, Oral cancer, Radiotherapy

## Abstract

**Background:**

The aim of this retrospective observational study was to evaluate toxicities, overall survival, and locoregional control in elderly oral squamous cell carcinoma patients who had undergone retrograde intra-arterial chemotherapy combined with radiotherapy.

**Methods:**

Thirty-one elderly patients over 80 years old with oral squamous cell carcinoma were enrolled in present study. The treatment schedule consisted of intra- arterial chemotherapy (docetaxel, total 60 mg/m^2^; cisplatin, total 150 mg/m^2^) and daily concurrent radiotherapy (total, 60 Gy) for 6 weeks.

**Results:**

The median patient age was 82.5 years old (range, 80–88 years). Of the 31 patients, six (19%) had stage II, 6 (19%) had stage III, 17 (55%) had stage IVA, and 2 (6%) had stage IVB. The median follow-up period for all patients was 37 months (range, 7–86 months). The 3-year overall survival and locoregional control rates were 78% and 81%, respectively. The major acute grade 3 adverse events were oral mucositis in 22 (71%) patients, neutropenia in 16 (52%), and dermatitis in 11 (35%). With respect to late toxicities, 1 patient (3%) developed grade 3 osteoradionecrosis of the jaw. No grade 4 or higher toxicities were observed during the treatment and follow-up periods.

**Conclusions:**

Retrograde intra-arterial chemotherapy combined with radiotherapy was effective in improving overall survival and locoregional control even for elderly patients.

## Introduction

The percentage of elderly patients with head and neck squamous cell carcinoma within the population is increasing as a result of the increased average age of the population. Almost 24% of patients with head and neck cancer are over the age of 70 years [1].

The majority of studies support appropriate surgical management of resectable head and neck malignancies (HNM) in elderly patients [2, 3]. With respect to the postoperative quality of life (QOL) of HNM patients, there were no significant differences between elderly and younger patients [4]. However, especially in oral cancer patients, it is obvious that some functions, such as speech, mastication, and swallowing, were more affected by the surgical intervention.

Several single-institutional studies have suggested reasonable rates of toxicities and excellent oncologic outcomes with the use of chemoradiotherapy (CRT) in elderly HNM patients. However, in such head and neck cancer clinical trials dealing with elderly patients, the age limit is often restricted to 65–75 years [5, 6]. Furthermore, in most large phase III trials evaluating concurrent CRT for HNM, the median age of enrolled patients has consistently been under 60 years [7]. In our institution, we have performed retrograde intra-arterial infusion chemotherapy via the superficial temporal artery (STA) and/or the occipital artery (OA) concurrent with radiotherapy for advanced HNM patients with good outcomes [8, 9]. However, there is no conclusive evidence to show the efficacy and safety of our treatment for elderly patients. The aim of the present study was to evaluate the clinical outcomes of elderly patients (over 80 years old) with oral squamous cell carcinoma (OSCC) treated with combined therapy of retrograde intra-arterial infusion chemotherapy concurrent with radiotherapy at a single institution.

## Material and methods

### Study design

This retrospective observational study was conducted between 2009 and 2015. In the present study, the patients met the following criteria: pathologic confirmation of OSCC; T stage of late T2-T4b with any N stage and no distant metastasis according to the 2002 UICC staging system [10]; Eastern Cooperative Oncology Group (ECOG) performance status (PS) of 0–2; age over 80 years; no active double cancer at the beginning of treatment, and no previous radiotherapy to the head and neck region; bone marrow function maintained, with a white blood cell count of at least 3500/mm^3^, a platelet count of at least 100,000/mm^3^, and a hemoglobin level of at least 9 g/dL; and no cerebral infarction or severe liver, kidney, heart, or lung dysfunction. In this study, the primary study outcome was overall survival (OS). Secondary measures included locoregional control (LRC) and treatment-related toxicities.

### Radiotherapy

Treatment planning for X-ray irradiation (XRT) was based on three-dimensional CT images taken at 2-mm intervals. The gross tumor volume (GTV) was determined by CT, MRI, and positron emission tomography (PET) CT scans prior to treatment. Clinical target volume (CTV) was defined as GTV plus a 5-mm margin. Planning target volume (PTV) was basically defined as CTV plus a 5-mm margin, but it could be finely adjusted where necessary to take into account organs at risk. The daily XRT fraction was 2 Gy using a 6-MV linear accelerator, and the XRT schedule was 60 Gy in 30 fractions delivered over 6 weeks (conventional technique). For the XRT of patients with no cervical lymph node metastases, the XRT field was set up to cover the primary lesion and prophylactically the level I–III lymph node region as the CTV. For patients with cervical lymph node metastases, the XRT field was set up to cover the primary tumors and the ipsilateral (levels I–IV for N1) or bilateral (levels I–V for N2) cervical lymph node areas, including lymph node metastases as the CTV. After a total dose of 40 Gy had been delivered to the initial field, an additional 20 Gy was delivered to the primary tumors and metastatic lymph nodes within the shrunken field.

### Retrograde intra-arterial infusion chemotherapy

Catheterization from the STA and OA was performed as previously reported [11–13]. In the case of a tumor lesion that involved the contralateral side beyond the median line, another catheter was inserted in the contralateral side for bilateral arterial injection.

**Fig. 1:**
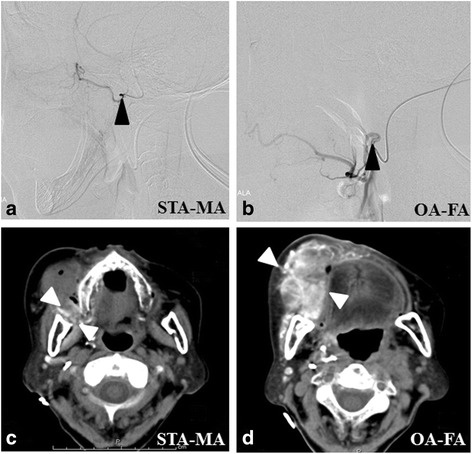
Digital subtraction angiograms (DSAs) and axial views of angio-CT images through retrograde intra-arterial infusion (carcinoma of the right buccal mucosa: T4aN0M0). **a**, **b** The catheters are selectively inserted into the right MA via the STA **a** and into the right FA via the OA **b** (*black arrow heads*: the tip of catheters). **c**, **d** *Axial view* of the angio-CT images after infusion of a small amount of contrast medium through the catheters. Tumor staining of the upper and posterior margin of the tumor is seen through the right MA (**c**: *white arrow heads*). Almost the whole area of the tumor is stained through the right FA. (**d**: *white arrow heads*). Abbreviations: MA, maxillary artery; STA, superficial temporal artery; FA, facial artery; OA, occipital artery

The tips of catheters were selectively inserted in the lingual artery (LA) in carcinoma of the tongue and the floor of the mouth, the facial artery (FA) in carcinomas of the tongue, the floor of the mouth, the buccal mucosa, and the lower and upper gingiva, and the maxillary artery (MA) in carcinomas of the hard palate and upper gingiva. After catheterization, the perfusion area of the anticancer agent was confirmed by digital subtraction angiography (DSA) and angio-CT. Angio-CT was performed with slow infusion via a catheter to determine whether the anticancer agents delivered via arterial infusion permeated the entire tumor (Fig. 1). Moreover, in the treatment period, it was confirmed that the arterial injection covered the tumor by infusion of indigotindisulfonate sodium more than once a week.

**Fig. 2:**
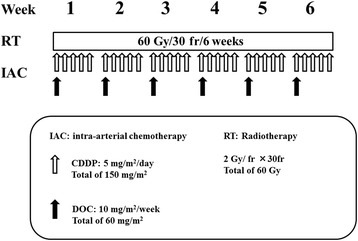
The treatment schedule consists of intra-arterial chemotherapy (docetaxel, total 60 mg/m^2^; cisplatin, total 150 mg/m^2^) and daily concurrent radiotherapy (total, 60 Gy) for 6 weeks

The anticancer agent was injected in a bolus over 1 h through the catheter during the irradiation. The dose of docetaxel (DOC) was 10 mg/m^2^/week, for a total of 60 mg/m^2^ during the whole treatment course, and that of cisplatin (CDDP) was 5 mg/m^2^/day, for a total of 150 mg/m^2^ (Fig. 2). Sodium thiosulfate (STS), a CDDP-neutralizing agent, was also administered intravenously at 1 g/m^2^ immediately after arterial infusion of CDDP.

### Evaluation and follow-up

The clinical response was evaluated in all enrolled patients using PET-CT, enhanced MRI, and CT imaging studies 4 weeks after the completion of the planned treatment schedule. The clinical response was judged according to the Response Evaluation Criteria in Solid Tumors guidelines (RECIST guideline, version 1.1).

Tissue biopsy of the primary lesion was also performed to evaluate the clinical response pathologically 5–7 weeks after the completion of the treatment schedule. If residual primary tumor was seen on tissue biopsy, salvage surgery was planned 8 weeks after completion of initial treatment. If the primary tumor was controlled and there were residual lymph node metastases after treatment, radical neck dissection was planned.

### Toxicity assessment

The acute and late adverse events during treatment and follow up period were evaluated according to the National Cancer Institute Common Terminology Criteria for Adverse Events version 4.0 (CTCAE v4.0) per protocol. Acute toxicity was defined as that occurring within 3 months of the completion of treatment. A complication that occurred more than 3 months after completion of treatment was considered to be a late toxicity. The evaluation categories of acute toxicities were blood cell counts, renal function, oral mucositis, radiation dermatitis, dysphagia, fever, and nausea. As late toxicities, dry mouth (xerostomia) and osteonecrosis of the jaw (osteoradionecrosis: ORN) were evaluated.

### Statistical analysis

OS was calculated from the start of treatment to the date of death. LRC was defined as lack of progressive disease at the primary tumor site and cervical lymph node metastases*.* The OS and LRC rates were calculated by the Kaplan-Meier method. In both univariate and multivariate analysis, the Cox’s proportional hazards model was used to analyze potential variables associated with survival and locoregional recurrence. For multivariate analysis, the variables with univariate significance of *P* ≤ 0.05 were tested. The results are shown as hazard ratios (HR), 95% confidence intervals (CI) and *P* values on the basis of the Cox’s proportional hazards model. All *P* values were two-sided; *P* < 0.05 was considered to indicate statistical significance. Statistical analysis was based on intent - to - treat manner.

## Results

### Patients’ characteristics

Between January 2009 and August 2015, a total of 31 elderly patients, over 80 years of age, with OSCC were enrolled in the present study. The median patient age was 82.5 years (range, 80–88 years). Of the 31 patients, 12 (39%) were men and 19 (61%) were women. The PS was 0 in 25 patients (81%), 1 in 4 patients (13%) and 2 in 2 patients (6%). Six patients (19%) had Stage II, 6 (19%) had Stage III, 17 (55%) had Stage IVA, and 2 (6%) had Stage IVB (Table 1).

**Table 1:** Patients’ characteristics

Characteristics	Values (%)
No. of patients	31 (100%)
Median age (years) (range)	82.5 (80–88)
Sex	
Male	12 (39%)
Female	19 (61%)
ECOG -PS	
0	25 (81%)
1	4 (13%)
2	2 (6%)
T classification	
T 1	0
T 2	6 (19%)
T 3	12 (39%)
T 4a	11 (35%)
T 4b	2 (6%)
N classification	
N 0	18 (58%)
N 1	5 (16%)
N 2b	7 (23%)
N 2c	1 (3%)
N 3	0
Stage	
II	6 (19%)
III	6 (19%)
IV A	17 (55%)
IV B	2 (3%)
Primary tumor site	
Tongue	12 (39%)
Upper gingiva	8 (26%)
Lower gingiva	5 (16%)
Buccal mucosa	4 (13%)
Floor of mouth	1 (3%)
Hard palate	1 (3%)

*ECOG* Eastern Cooperative Oncology Group, *PS* performance status

Values represent number of patients, except as otherwise stated

### Treatment delivery

The median total dose of XRT was 60 Gy (range, 42–70 Gy). The median cumulative CDDP and DOC doses were 150 mg/m^2^ (range, 105–175 mg/m^2^) and 60 mg/m^2^ (range, 50–70 mg/m^2^), respectively. Overall, 23 patients (74%) completed the planned course of CRT without discontinuation of chemotherapy, change of chemotherapy regimen, or radiation delay of more than 5 days. Eight patients (26%) had a treatment delay between 5 and 30 days (median, 7 days) secondary to nephrotoxicity. Catheter-related infection was seen in 3 patients (9%), and they were not able to complete the planned treatment schedule.

### Response and survival

**Fig. 3:**
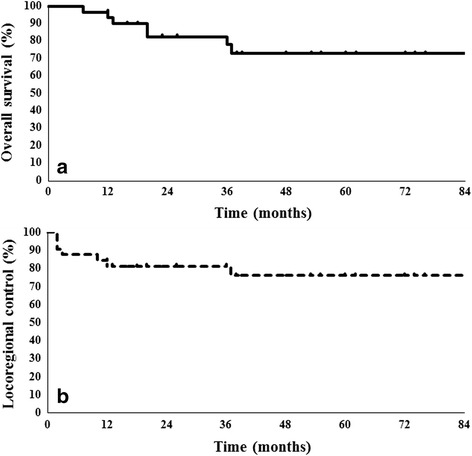
Overall survival rate **a** and locoregional control rate **b** using the Kaplan-Meier method. The 3-year overall survival rate and locoregional control rate are 78% and 81%, respectively

The median follow-up period for all patients was 37 months (range, 7–86 months). A complete response (CR) was achieved in 25 patients (81%), with a partial response (PR) in 6 patients (19%) at the primary tumor site. Two patients (6%) required radical neck dissection because of residual lymph node metastases in the follow-up period. Salvage surgery was performed in 1 patient (3%) who had residual primary tumor on tissue biopsy. A total of 7 patients (22%) died during the follow-up period: 3 patients (10%) died of lung metastasis, 2 patients (6%) died of local failure, 1 patient (3%) died of cervical neck failure, and 1 patient (3%) died of other causes. The 3-year OS and LRC rates were 78% and 81% respectively (Fig. 3).

### Toxicity

The major acute grade 3 adverse events were as follows: oral mucositis in 22 (71%) patients, neutropenia in 16 (52%), and dermatitis in 11 (35%). A total of 22 patients (71%) experienced grade 3 dysphagia and needed feeding tube (percutaneous endoscopic gastrostomy tube) placement during the treatment period. Severe mucositis and dysphagia were likely to recover within 8 to 12 weeks after treatment to the extent that oral intake was possible. As late toxicities, 1 patient (3%) experienced grade 3 ORN of the jaw. During the treatment period, severe complications, such as neurological complications, were not encountered. No grade 4 or higher toxicities were observed during the treatment and follow-up periods (Table 2).

**Table 2:** Treatment-related acute and late toxicities

	No, of patients by toxicity grade (%)				
	Grade 1	Grade 2	Grade 3	Grade 4	Grade 5
Acute					
Neutropenia	9 (29%)	6 (19%)	16 (52%)	0	0
Thrombocytopenia	11 (35%)	8 (26%)	12 (39%)	0	0
Anemia	6 (19%)	19 (61%)	6 (19%)	0	0
AKI	10 (32%)	4 (13%)	0	0	0
Fever	18 (58%)	7 (23%)	0	0	0
Mucositis oral	0	9 (29%)	22 (71%)	0	0
Dermatitis	0	20 (65%)	11 (35%)	0	0
Dysphagia	0	9 (29%)	22 (71%)	0	0
Late					
Dry mouth	15 (48%)	13 (42%)	0	0	0
Osteoradionecrosis	2 (6%)	7 (23%)	1 (3%)	0	0

*Abbreviations: CTCAE v 4.0* Common Terminology Criteria for Adverse Events version 4.0, *AKI* Acute kidney injury

### Factors related to survival and locoregional recurrence

In the univariate analysis, ECOG-PS and treatment delay (more than 5 days) had a significant impact on locoregional recurrence, and there were no significant variables associated with survival (Table 3). In the multivariate analysis using these two variables, ECOG-PS had a significant impact on locoregional recurrence (Table 4).

**Table 3:** Univariate analysis for patient’s characteristics and treatment factors

Variables	Level	No of patients	Survival			Locoregional recurrence		
			HR^b^	95% CI^c^	*P* value	HR	95% CI	*P* value
Age	<83	20	1		0.495	1		0.438
	≥83	11	1.333	0.334–11.875		2.098	0.776–18.226	
Sex	Male	12	1		0.534	1		0.402
	Female	19	1.785	0.287–11.128		2.561	0.284–23.047	
ECOG-PS	0	25	1		0.091	1		0.026*
	≥1	6	5.250	0.766–35.978		9.265	1.331–44.532	
T classification	2 or 3	18	1		0.149	1		0.066
	≥4a	13	2.258	0.745–6.839		4.084	0.911–18.303	
N classification	0 or1	23	1		0.632	1		0.435
	≥2b	8	1.240	0.513–2.996		2.223	0.298–16.559	
Stage	II, III	12	1		0.237	1		0.083
	≥IVA	19	2.075	0.618–6.968		7.147	0.776–56.792	
Total dose of RT^d^	≥60 Gy	20	1		0.208	1		0.435
	<60 Gy	11	0.233	0.024–2.252		1.575	0.987–25.154	
Treatment delay^a^	No	19	1		0.264	1		0.044*
	Yes	12	2.667	0.477–14.904		7.965	1.052–45.271	

*P < 0.05; ^a^Treatment delay, more than 5 days; ^b^hazard ratio; ^c^confidence interval; ^d^radiotherapy

**Table 4:** Multivariate analysis for patient’s characteristics and treatment factors

Variables	Level	No of patients	Locoregional recurrence		
			HR^b^	95% CI^c^	*P* value
ECOG-PS	0	25	1		0.044*
	≥1	6	9.954	1.058–69.672	
Treatment delay^a^	No	19	1		0.675
	Yes	12	1.610	0.173–15.011	

*P < 0.05; ^a^Treatment delay, more than 5 days; ^b^hazard ratio; ^c^confidence interval

## Discussion

Surgery can be effective in elderly HNM patients if the primary tumor could be excised without causing severe postoperative dysfunction [14]. Airoldi et al. [15] reported the treatment outcome of performing surgery with postoperative CRT for elderly HNM patients (median age, 73.5 years [range, 70–78 years]). According to their report, the 3-year OS and Local control were 64% and 79%, respectively. However, major surgery is less frequently offered to elderly patients because the incidence of medical complications is significantly increased due to the presence of degenerative conditions and comorbidities [16]. Derks et al. [17] evaluated the frequency of postoperative complications in elderly HNM patients. According to their report, complications of major surgery, such as pneumonia, dehydration, and feeding disturbance, occurred in 53% of patients 80 years and older group. With respect to the postoperative QOL questionnaire in elderly patients with locally advanced HNM undergoing major head and neck surgery, Khafif et al. [18] reported that oral functions such as “chewing” and “speech” were decreased by the surgical intervention more remarkably in the elderly patients group than in the younger group.

Non-surgical therapy, radiotherapy (RT) is often preferred in the treatment of elderly HNM patients. The most commonly used method in elderly patients is conventional fractionation (fr) of 1.8–2.0 Gy/fr, with a total dose of 70 Gy [19]. Schofield et al. [20] reported the clinical outcomes of definitive RT for patients aged over 80 years. The 5-year OS was 28%, and the outcomes were poor. According to the meta-analysis by Pignon et al. [21], which analyzed the synergy of systemic chemotherapy in combination with RT in HNM, there was no survival benefit from CRT in elderly HNM patients.

Altered fractionation, such as accelerated RT, and hyperfractionated RT techniques were designed to improve effectiveness by delivering more than one fraction per day with a reduced dose per fraction, but an increased total dose, aiming to increase the likelihood of LRC by reducing affected tumor cell recovery and shorten the hospitalization period [22]. However, according to a randomized trial by Jackson et al. [23], an increased risk of late toxicities was observed with accelerated fractionation without total dose reduction compared with conventional fraction. Furthermore, according to a meta-analysis of randomized trials, patients over the age of 70 years did not benefit from hyperfractionated RT compared with conventional RT (HR, 1.08) [24].

In recent decades, some studies of combined therapy of intra-arterial chemotherapy and RT have shown good treatment outcomes. In our clinical study, which included 112 oral cancer patients with advanced stage disease, we reported good treatment outcome (3 and 5-year OS rates of 74.6%, and 71.3%, respectively) with relatively low prescription total doses of 60 Gy by combining intra-arterial infusion chemotherapy [9]. In the present study, the prescription total RT dose was 60 Gy, and good OS and LRC were achieved. Even for elderly patients, our treatment modality might be effective in improving prognosis.

Recently, cetuximab has been used as a molecular targeting agent in the treatment of inoperable HNM patients [25, 26]. In some trials, cetuximab and/or RT demonstrated better OS and LRC rates in young HNM patients to some extent [26]. However, these results were not able to determine the efficacy and safety of cetuximab in elderly HNM patients. The recent report of the Bonner et al. [26] study demonstrated, on subgroup analysis, that cetuximab had no benefit in patients aged 65 years or older. To date, no age-specific trials have clarified the value of cetuximab in elderly patients.

CDDP has been commonly used in the treatment of HNM. Furthermore, Yabuuchi et al. [27] reported better clinical outcomes for HNM in their intra-arterial chemotherapy study by combined CDDP and DOC than by CDDP alone. However, CDDP has severe side effects such as nephrotoxicity. In the present study, more than half of the patients experienced severe bone marrow suppression due to poor hematopoietic function. However, most of them completed the planned treatment schedule by proper use of granulocyte colony stimulating factor (G-CSF) for the purpose of improving neutropenia, febrile neutropenia, and sepsis. Furthermore, severe kidney dysfunction was not observed. This is because the median cumulative CDDP dose in the present study was relatively low compared with that of general systemic chemotherapy using CDDP. These results suggest that the present treatment of intra-arterial chemotherapy combined with RT is safe and acceptable even for elderly OSCC patients.

As a late toxicity, grade 2 xerostomia developed in 13 patients (42%). An earlier report indicated that sparing an overall low dose to the parotid gland could lead to less parotid gland hypofunction and xerostomia [28]. In order to avoid low and high doses to the entire parotid gland, particle beam therapy, such as proton beam therapy (PBT), could be used to minimize the exposure to salivary gland tissue. Eight patients (26%) also developed over grade 2 ORN as another late adverse event. In general, the incidence of ORN post RT or intensity-modulated radiotherapy (IMRT) was reported to range from 2% to 16% [29, 30]. Takayama et al. [31] reported good treatment outcomes of PBT combined with intra-arterial chemotherapy for T3–4 tongue cancer (3-year OS of 87%). They also reported that, by using a spacer to fix the tongue and to avoid extra irradiation to the mandible, the incidence of ORN was only 3% (1 of 33 patients). By applying PBT and a spacer, xerostomia and ORN might be more effectively prevented.

## Conclusions

Retrograde intra-arterial chemotherapy combined with RT was effective in improving OS and LRC even for elderly OSCC patients. The outcome was not inferior to the past studies investigating the results of surgery. It is true that many patients experienced severe bone marrow suppression in the present study, but no treatment-related deaths or neurological complications were observed. The observed toxicities were tolerable and manageable. Late adverse events such as ORN should be monitored continuously in the future.

## Abbreviations

CDDP: Cisplatin
CRT: Chemoradiotherapy
DOC: Docetaxel
DSA: Digital subtraction angiography
FA: Facial artery
G-CSF: Granulocyte colony stimulating factor
HNM: Head and neck malignancies
LA: Lingual artery
LRC: Locoregional control
MA: Maxillary artery
OA: Occipital artery
ORN: Osteoradionecrosis
OSCC: Oral squamous cell carcinoma
STA: Superficial temporal artery
STS: Sodium thiosulfate
XRT: X-ray irradiation
